# Salvianolic acid A alleviates lipopolysaccharide-induced disseminated intravascular coagulation by inhibiting complement activation

**DOI:** 10.1186/s12906-022-03720-z

**Published:** 2022-09-20

**Authors:** Qi-Yun Zhang, Jing Guo, Lin Xu, Ying Wei, Shu-Ting Zhou, Qing-Yu Lu, Li Guo, Qian-Yun Sun

**Affiliations:** 1 State Key Laboratory of Functions and Applications of Medicinal Plants, Guizhou Medical University, Guiyang, 550014 China; 2 Center for Pharmacology and Bioactivity Research, The Key Laboratory of Chemistry for Natural Products of Guizhou Province and Chinese Academy of Sciences, Guiyang, 550014 China

**Keywords:** Salvianolic acid A, Disseminated intravascular coagulation, Lipopolysaccharide, Complement system, C3b

## Abstract

**Introduction:**

Disseminated intravascular coagulation (DIC) is a syndrome characterized by coagulopathy, microthrombus, and multiple organ failure. The complement system in DIC is overactivated, and the functions of complement and coagulation pathways are closely related. Our previous screening revealed that salvianolic acid A (SAA) has anti-complement activity. The hyper-activated complement system was involved in the lipopolysaccharide (LPS) induced DIC in rats. The effects of SAA anti-complement action on LPS-induced DIC in rats were investigated.

**Methods:**

The complement activity of the classical pathway and alternative pathway was detected through an in vitro hemolysis assay. The binding sites of SAA and complement C3b were predicted by molecular docking. LPS-induced disseminated coagulation experiments were performed on male Wistar rats to assess coagulation function, complement activity, inflammation, biochemistry, blood routine, fibrinolysis, and survival.

**Results:**

SAA had an anti-complement activity in vivo and in vitro and inhibited the complement activation in the classical and alternative pathway of complement. The infusion of LPS into the rats impaired the coagulation function, increased the plasma inflammatory cytokine level, complemented activation, reduced the clotting factor levels, fibrinogen, and platelets, damaged renal, liver, and lung functions, and led to a high mortality rate (85%). SAA treatment of rats inhibited complement activation and attenuated the significant increase in D-dimer, interleukin-6, alanine aminotransferase, and creatinine. It ameliorated the decrease in plasma levels of fibrinogen and platelets and reversed the decline in activity of protein C and antithrombin III. The treatment reduced kidney, liver, and lung damage, and significantly improved the survival rate of rats (46.2 and 78.6% for the low- and high-dose groups, respectively).

**Conclusion:**

SAA reduced LPS-induced DIC by inhibiting complement activation. It has considerable potential in DIC treatment.

## Introduction

Disseminated intravascular coagulation (DIC) refers to the activation of platelets or coagulation factors under the action of certain pathogenic factors, and a large amount of soluble procoagulant substances enters the blood, thereby causing a pathological process characterized by coagulation dysfunction [1, 2]. The causes of DIC include infection, malignancy, extensive tissue trauma, cardiopulmonary bypass, and obstetric accidents [1–5]. DIC usually leads to the formation of many microthrombi, leading to circulatory and other visceral dysfunction, consumptive coagulopathy, secondary fibrinolysis, and clinical manifestations, such as shock, hemorrhage, embolism, and hemolysis [1, 2]. Bacterial infection can lead to DIC. Bacteria and their toxins can damage tissues and vascular endothelial cells, activate factor XII kallikrein and bradykinin, activate the coagulation system, and lead to disturbances in the balance of coagulation and anticoagulation, and eventually induce DIC [3, 6, 7]. DIC is a late complication of sepsis, runs through the entire pathological process of sepsis, and is among the critical links in sepsis development and prognosis [8].

The early phases of sepsis are associated with a strong activation of the three complement pathways (classical, alternative, and lectin), generating many potent pro-inflammatory peptides [9–11]. Complement activation varies considerably in different groups of sepsis patients and is higher in patients with DIC than in those without DIC. Complement 3a, membrane attack complex (MAC), and mannan binding lectin (MBL) significantly increase in septic patients with DIC [12]. Meanwhile, the degree of complement activation is related to DIC, severity, intensive interventions, and mortality [13].

The interaction between the complement and coagulation systems in DIC has been frequently reported in previous research [14–16]. Complement system activation affects coagulation in multiple ways [17]. A key enzyme in the activation of the lectin pathway (LP) (MASP-2) can generate thrombin by direct cleavage of prothrombin [18]. Similarly, even in the absence of factor V (FV), the C5b-9 complex, the terminal component of complement activation, has a negative effect on prothrombin [19]. C3a and C5a bind to C3a and C5a receptors on platelets and rapidly activate platelet function [19, 20]. Inserting the membrane attack complex (C5b-9) into platelets, leading to platelet activation and degranulation [15, 19]. Both the membrane attack complex (MAC) with glycolysis and the C5b-9 terminal complement complex without cytolysis mediate procoagulant activity by inducing the expression of tissue factor (TF) in vascular endothelial cells [21–23]. At the same time, C5b-9 induces the eversion of phosphatidylserine on the platelet surface, providing a catalytic surface for prothrombinase assembly [24]. Anaphylatoxin C5a increases coagulation activity through various effects on cells; C5a can induce endothelial cell and neutrophil TF expression [25, 26]. C5a can induce a shift in basophil and mast cell activity from fibrinolysis to thrombosis by upregulating plasminogen activator inhibitor 1 (PAI-1) [14]. Some complement inhibitors, such as Compstatin [27] and RA101295 [28], reportedly improve consumptive coagulation responses and protect organ function in sepsis models. Therefore, inhibition of complement activation is a therapeutic strategy for ameliorating sepsis-induced DIC.

Chinese herbs are widely used to treat thrombotic diseases throughout history. The dried root or rhizome of *Salvia miltiorrhiza* is a treatment used for cardiovascular diseases [29]. Salvianolic acid A (SAA), one of the major water-soluble compounds of *S. miltiorrhiza*, is the most bioactive component of salvianolic acids [30]. Studies show that SAA inhibits platelet aggregation [31] and is antithrombotic [31], antioxidative [32], and anti-inflammatory [33]; it has other comprehensive pharmacological effects. Moreover, our previous screening showed that SAA has an anti-complement activity. However, its ameliorating effect on LPS-induced DIC in rats has not been investigated. Therefore, this study aimed to examine the effect of SAA on LPS-induced DIC and the mechanism of action against complement.

## Materials and methods

### Reagents

SAA (CAS: 96574–01-5, Cat No.PS1113, purity: 99.0%) was purchased from PUSH BIO-TECHNOLOGY Co., Ltd. (Chengdu, China). The SAA solution was freshly prepared before use. Lipopolysaccharide (LPS, *Escherichia coli* O111:B4 endotoxin, Cat No. L4130) was purchased from Sigma (St Louis, USA). TNF-α (Cat No. F16960), IL-1β (Cat No. F15810), IL-6 (Cat No. F15870), IL-8 (Cat No. F15880), C3 (Cat No. F15251), protein C (Cat No. F16520), and D-dimer (Cat No. F15300) enzyme-linked immunosorbent assay (ELISA) kits were purchased from RapidBio Lab (Shanghai, China). The reagent packs for the activity assays of antithrombin III (ATIII)(Cat No.221122) were obtained from Hyphen BioMed Company (Neuville-sur-Oise, France). The chromogenic substrate S2251 (Cat No.B2251) was purchased from Shanghai Boatman Biotechnology Co., Ltd. (Shanghai, China). Sheep blood (Cat No. SBJ-RBC-S003) and hemolysin (Cat No. SBJ-RXS10) were from SenBeiJia Biological Technology Co., Ltd. (Nanjing, China). Rabbit blood cells were collected in Alsevers’ solution. All other reagents were of analytical grade and obtained from commercial sources.

### Animals

Male Wistar rats (250–280 g, SPF II Certificate; No. SCXK 2020–0001) and adult male New Zealand white rabbits (weight: 2–2.5 kg, SPF II Certificate; No. SCXK 2020–0001) were purchased from Liaoning Longevity Biotechnology Company Limited (Liaoning, China). Before the experiment, the animals were fed standard rodent chow and water and monitored at a controlled temperature and under a 12 h light/12 h dark cycle for 5 days. The experiments were performed in accordance with the protocols approved by the Laboratory Animal Ethics Committee of Guizhou Medical University (No. 2101032).

### Normal human serum (NHS) preparation

The 5 mL of blood was drawn from each of the three normal healthy volunteers in the research group, and after standing at room temperature for 1 h, centrifuged at 3000 rpm for 15 min, and then the serum was taken, mixed, and dispensed. The study protocol was in compliance with Helsinki Declaration. The volunteers provided written informed consent to confirm the blood sample was used only in this study.

### Complement activity assay

Anticomplement activity detection of the classical pathway (CP) [34] was performed by incubating GGVB +  + diluted sample solution (100 µL) with 100 µL GGVB +  + diluted serum and 100 µL 2% sensitized sheep erythrocytes at 37 °C for 30 min. Then, the reaction mixture was centrifuged at 3000 r/min for 5 min. Next, 200 µL supernatant was obtained. The optical density (OD) value was measured at 412 nm to calculate the hemolysis rate.

Anticomplement activity detection of the alternative pathway (AP) [34]. GVB-Mg-EGTA diluted sample solution (100 µL) was incubated with 100 µL GVB-Mg-EGTA diluted serum and 100 µL rabbit erythrocytes at 37 °C for 30 min. Then, the reaction mixture was centrifuged at 3000 r/min for 5 min. Next, 200 µL supernatant was obtained, and the OD value at 412 nm was measured to calculate the hemolysis rate.

The sample control of each dilution, blank control, and 100% lysis control were prepared under the same conditions. The corrected absorbance of each dilution of the samples was obtained by subtracting the absorbance of the sample control from each value.

### Inhibition of the classical complement pathway C3/C5 convertase formation test

According to a method used in a previous study [35], each sample solution and rat serum (100 µL) were placed in a test tube, incubated in a water bath at 37 °C for 10 min, mixed with 100 µL sensitized sheep erythrocytes, and set at 37 °C for 3 min. Then, 1 mL normal saline was used to wash the red blood cells. The samples were centrifuged at 2,000 r/min for 10 min, the supernatant was aspirated, and 100 µL rat serum was added. This was diluted with GVB buffer containing 0.04 mol/L ethylenediaminetetraacetic acid (EDTA; 1:5) at 37 °C. The samples were incubated in a water bath for 30 min, and the reaction was stopped with 0.9 mL of saline. The samples were centrifuged at 2000 r/min for 10 min, the supernatant was obtained, and the absorbance was measured at 412 nm.

### Molecular docking

The 3D structure of C3b came from the RCSB PDB database (PDB ID: 2I07) and other complexes and water molecules in the protein were removed by Discovery Studio 2016 visualizer (DS) software. The 3D structure of SAA was retrieved from PubChem (CID: 5,281,793) using Chem3DUltra14.0 SAA for the format conversion. According to the method used in other studies [36, 37], the ProteinsPlus protein site prediction platform was used to predict the binding site of the two to obtain the potential binding site of the target. Molecular docking simulation software (AutoDock 4.2. 6) was used to hydrogenate C3b and calculate the Gasteiger charge while operating on SAA; molecular semi-flexible docking was performed after completion, and local search parameters were used as the algorithm to obtain the docking result [38–40]. PyMOL software was used to construct the docking result [41]. Finally, the conformation with the minimum binding energy was selected from the binding conformations obtained by docking for subsequent analysis. A molecular simulation software (Discoverystudio) was used to create diagrams.

### Experimental models and drug treatments

LPS-induced in vivo model of complement activation in rats. The 20 rats were randomly divided into five groups (*n* = 4/group), as follows: (1) normal group: normal rat; (2) LPS-1 h group: LPS induction for 1 h; (3) LPS-3 h group: LPS induction for 3 h; (4) LPS-6 h group: LPS induction for 6 h; and (5) LPS-9 h group: LPS induction for 9 h. The 15 mg/kg LPS was injected into the tail vein of the animals except to the animals in the normal group. After 1, 3, 6, and 9 h, rats were sacrificed via intraperitoneal (i.p.) injection of pentobarbital sodium (50 mg/kg), and blood was collected for further assays.

The effects of SAA on LPS-induced complement activation in rats were determined. The 25 rats were randomly divided into five group (*n* = 5/group): (1) control group: normal saline + normal saline; (2) LPS group: normal saline + 15 mg/kg LPS; (3) SAA80 group: 80 mg/kg SAA + 15 mg/kg LPS; (4) SAA40 group: 40 mg/kg SAA + 15 mg/kg LPS; and (5) SAA20 group: 20 mg/kg SAA + 15 mg/kg LPS. The control and LPS groups received intraperitoneal (i.p.) injections with the same volume of normal saline. The SAA groups were intraperitoneally injected with SAA at 80, 40, and 20 mg/kg. One hour after the administration, the control group was injected with normal saline through the tail vein, and the other groups were injected with 15 mg/kg LPS through the same route. After 3 h of LPS induction, rats were sacrificed by an intraperitoneal (i.p.) injection of pentobarbital sodium (50 mg/kg), and blood was collected for further assays.

The effects of SAA on complement and coagulation function in normal rats were determined. The 20 rats were randomly divided into four groups (*n* = 5/group), as follows: (1) normal group: normal rat; (2) SAA-1 h group: SAA treatment for 1 h; (3) SAA-2 h group: SAA treatment for 2 h; and (4) SAA-4 h group: SAA treatment for 4 h. The 40 mg/kg SAA was intraperitoneally injected into the animals, except to those in the normal group. After 1, 2, and 4 h, rats were sacrificed by an intraperitoneal (i.p.) injection of pentobarbital sodium (50 mg/kg), and blood was collected for further assays.

The effects of SAA on LPS-induced death in rats were determined. The 36 rats were randomly divided into three groups (*n* = 13/group), as follows: (1) LPS group: normal saline + 15 mg/kg LPS; (2) SAA40 group: 40 mg/kg SAA + 15 mg/kg LPS; and (3) SAA20 group: 20 mg/kg SAA + 15 mg/kg LPS. The LPS group received an injection with the same volume of normal saline, and the SAA groups received 40 and 20 mg/kg SAA. One hour after the administration, 15 mg/kg LPS was injected into the tail vein to observe rat survival. Rats were sacrificed after 168 h by intraperitoneal (ip) injection of sodium pentobarbital (50 mg/kg).

The establishment of the DIC rat model was performed in accordance with methods described in the literature [42]. The 33 rats were randomly divided into three groups (*n* = 11/group), as follows: (1) control group: normal saline + normal saline; (2) LPS group: normal saline + 15 mg/kg LPS; and (3) SAA40 group: 40 mg/kg SAA + 15 mg/kg LPS. The control and LPS groups were intraperitoneally injected with the same volume of normal saline. The SAA group was intraperitoneally injected with SAA at 40 mg/kg. One hour after the administration, the control group was injected with normal saline through the tail vein, and the other groups were injected with 15 mg/kg LPS. After 3 h of LPS induction, rats were sacrificed by an intraperitoneal (i.p.) injection of pentobarbital sodium (50 mg/kg), and blood was collected for further assays.

### Blood specimen collection and detection

The rats were sacrificed by the i.p. injection of pentobarbital sodium (50 mg/kg). Thereafter, blood was withdrawn from rats via the insertion of a catheter into the abdominal aorta for blood collection in 3.8% sodium citrate (1:9 v/v citrate/blood), EDTA anticoagulant tube, and vacuum blood collection tube. The specimens were centrifuged at 3,000 rpm for 10 min and stored at − 80 ℃ until the assay.

Thromboelastography (Lepu Medical Cfmslepu-8800, China) was used to measure the coagulation function of blood. An automatic analyzer (Rayto Chemray 800, China) was utilized to measure the serum levels of alanine aminotransferase (ALT) and creatinine (Cre). A blood cell analyzer (Beckman Coulter DxH 600, USA) was used to detect complete blood count. The concentrations of TNF-α, IL-1β, IL-6, IL-8, C3, protein C, and D-dimer in the plasma were determined with an ELISA kit. The activities of ATIII were measured in accordance with the reagent pack instructions based on chromogenic substrates. The plasmin concentrations of plasma were determined with chromogenic substrate S2251.

### Thromboelastography test

Referring to the literature method [43], arterial blood samples for the kaolin thrombelastogram analysis were drawn from an arterial line into a tube containing 3.2% sodium citrate and analyzed by recalcification with 20 µL 0.2 M CaCl_2_. Five thrombelastogram variables were recorded: reaction time (R), k-time (K), maximum amplitude (MA), angle (α), and the calculated coagulation index (CI) value. R was the time until initial fibrin formation. K and α referred to the speed and kinetics of clot formation, respectively. MA denoted the maximal clot strength and stability of the fibrin clot, and CI was calculated from the MA, thereby providing a comprehensive parameter for coagulation function.

### Pathological tissue section

Fresh liver, kidney, and lung tissue samples were fixed with 4% paraformaldehyde, embedded in conventional paraffin, and cut into approximately 5 μm thick sections. Six paraffin sections were randomly taken from tissue samples and stained with hematoxylin and eosin. The morphological changes in the liver, kidney, and lung tissues of each group of rats were observed and photographed under a light microscope. Following the recommendations of the American Thoracic Society Official workshop report [44, 45], the stained lung tissue sections were used to assess lung injury under a microscope according to the following scoring criteria: ①Edema: 0 point represents non-existent, 1 point represents mild (10% alveolar involvement), 2 point represents moderate (10–50% alveolar involvement) or 3 point represents severe (50% alveolar involvement); ②Inflammation: 0 point represents none, 1 point represents mild (10 inflammatory cells/each high power field), 2 point represents moderate (10–50 inflammatory cells/each high power field), or 3 point represents severe (50 inflammatory cells/each high power field); ③Thickening of alveolar septae: 0 point represents none, 1 point represents mild, 2 point represents moderate, or 3 point represents severe; ④Alveolar epithelial injury (cell death, epithelial denudation, or Type II alveolar epithelial cell proliferation): 0 point represents absence, 1 point represents presence.

### Statistical analysis

Statistical analyses of data were performed using SPSS 18.0. The data were expressed as mean ± standard deviation (SD). Differences between groups were analyzed using one-way analysis of variance (ANOVA) and two-tailed t-tests. *P* < 0.05 was considered significant.

## Results

### In vitro anti-complement effect

**Fig.1:**
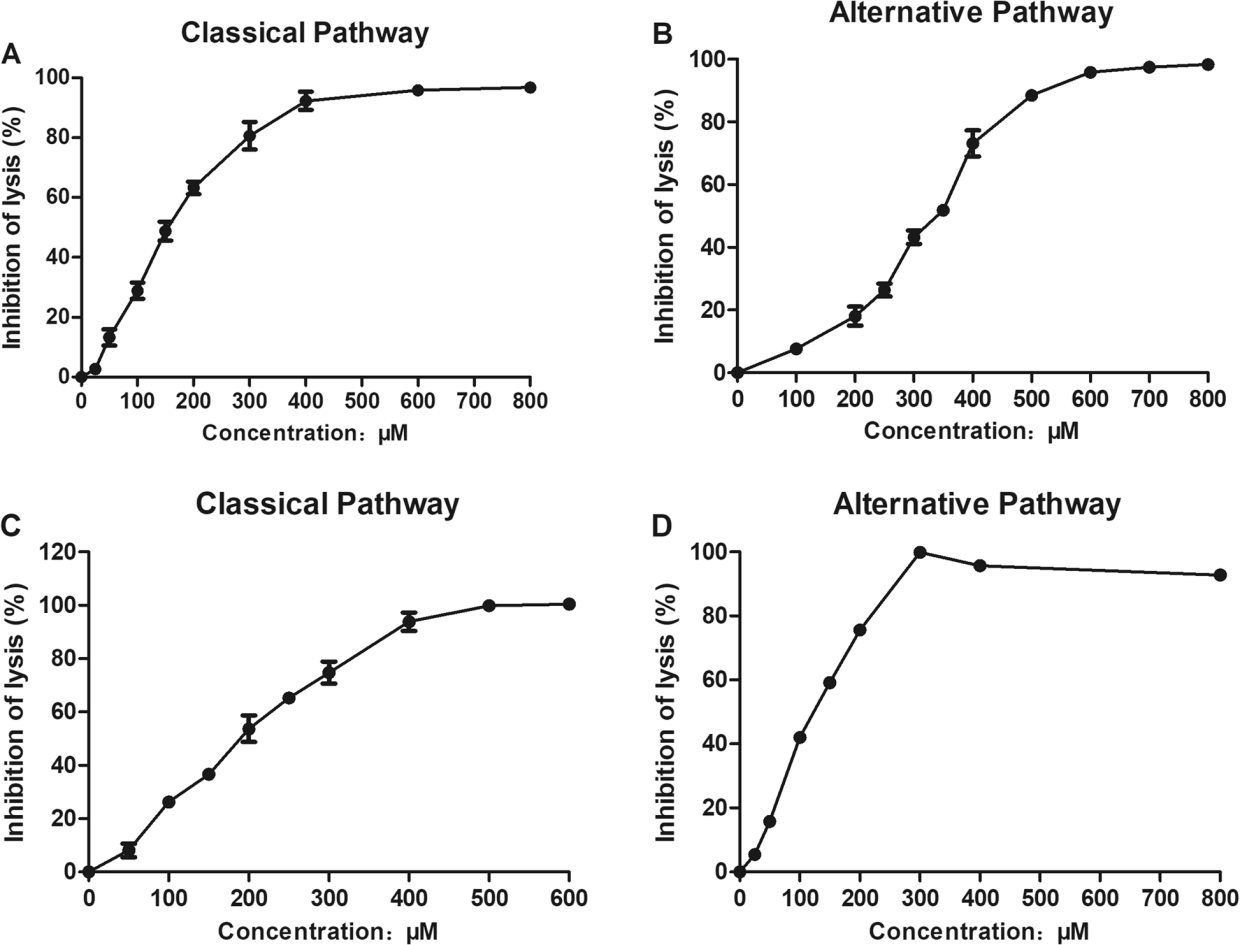
In vitro anti-complement activity of SAA. Inhibition of SAA on the classical pathway (**A** and **C**) and alternative pathway (**B** and **D**) of the complement system. **A** and **B** are the rat serum; **C** and **D** are the NHS. Results are expressed as percent inhibition of hemolysis. Data were presented as mean ± SD (*n* = 3)

SAA significantly inhibited hemolysis in the classical pathway (CP) and alternative pathway (AP) induced by rat serum (Figs. 1A and B, respectively). The CH_50_ and AP_50_ values were 152.12 ± 5.24 and 324.89 ± 8.32 µM (*n* = 3), respectively. SAA significantly inhibited hemolysis in the CP and AP induced by NHS (Figs. 1C and D, respectively). The CH_50_ and AP_50_ values were 193.27 ± 5.19 and 117.45 ± 3.28 µM (*n* = 3), respectively. SAA may inhibit the common protein of the CP and AP.

### Prediction of the targets of SAA in the complement activation cascade

**Fig.2:**
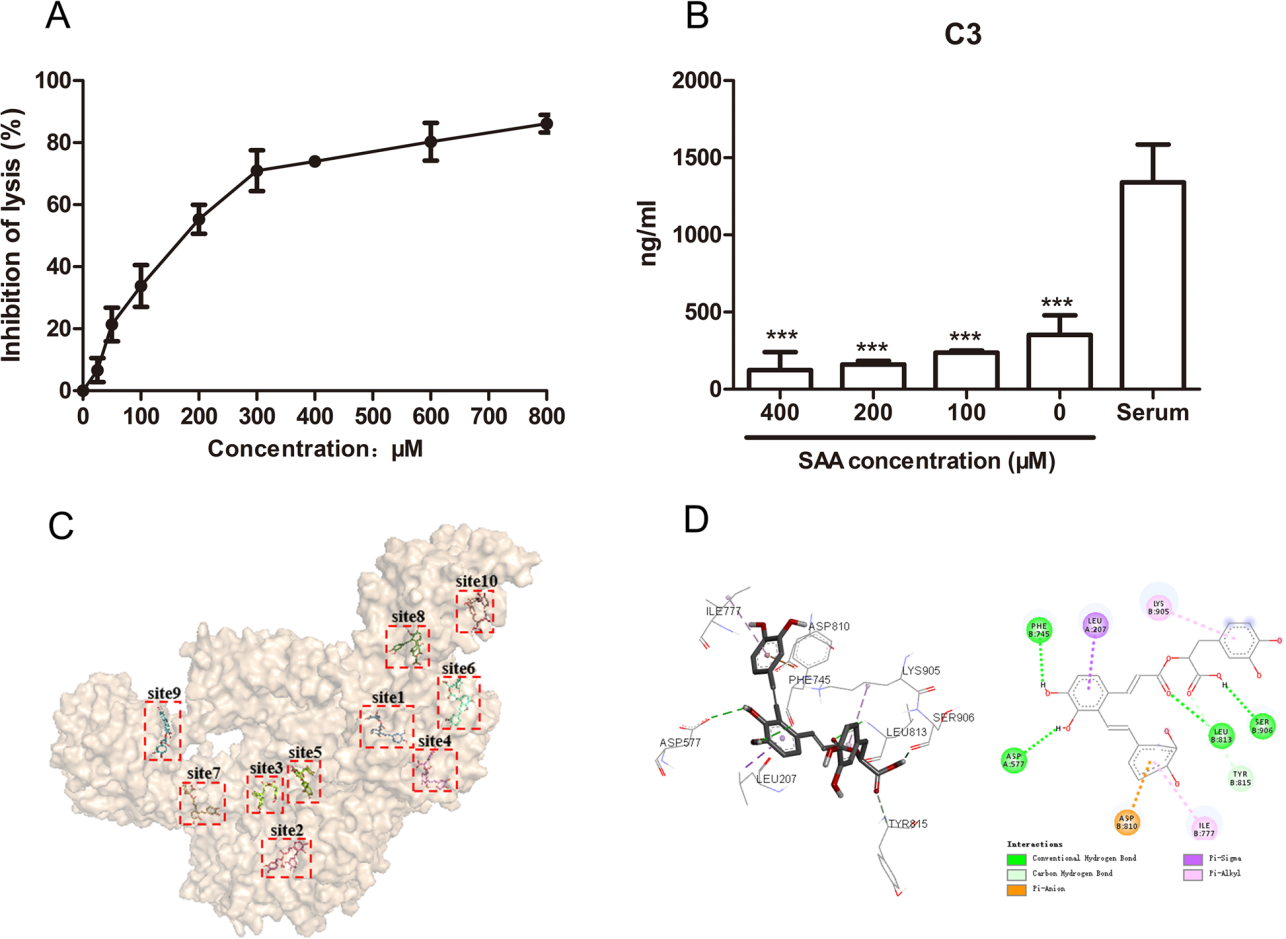
Prediction of the targets of SAA. **A** Effect of SAA on the formation of C3/C5 convertase of the complement classical pathway. Different concentrations of SAA were incubated with rat serum and sheep red blood cells for 3 min; after the sheep red blood cells were washed, rat serum containing 0.04 mol/L EDTA was added to initiate hemolysis. The absorbance was measured at 412 nm. Data were presented as mean ± SD (*n* = 4). **B** Contents of complement component C3 in the supernatant of classical pathway hemolysis experiment. Different concentrations of SAA were incubated with rat serum and sheep red blood cells for 30 min, centrifuge was performed to obtain the supernatant, and ELISA detection of the C3 content was performed. Data were presented as mean ± SD (*n* = 3). ***P < 0.001 compared with the serum group, statistical tests used were t-tests. **C** Map of 10 conjugation sites of SAA and C3b protein. **D** Conjugation diagram of SAA and C3b protein site 1

The complement classical pathway C3/C5 convertase formation experiment was used to detect the inhibitory effect of SAA. SAA was incubated with sensitized sheep red blood cells and rat serum to form C3/C5 convertase. The red blood cells were washed and reacted with the rat serum containing EDTA. Given that the serum containing EDTA can no longer generate new C3/C5 convertase, only the previously formed C3/C5 convertase can stimulate the hemolysis reaction of the classical pathway of the complement. The degree of hemolysis was positively correlated with the amount of C3/C5 convertase. Therefore, this functional experiment can prove whether SAA affects the formation of C3/C5 convertase. SAA significantly inhibited the classical-pathway C3/C5 convertase formation and showed a certain dose dependence (Fig. 2A).

The C3 content of the classical pathway hemolysis experiment in the rat serum was detected. C3 in the serum was consumed entirely (*P* < 0.001), and this event was independent of the inhibitory effect of SAA on hemolysis (Fig. 2B). SAA did not cause the inhibition of the CP when C3 convertase was formed, thereby converting the inhibition enzyme C5, which may inhibit the common protein C3b in the CP and AP.

SAA and C3b protein were molecularly docked. The binding energies of 10 potential sites were between -3.3 and -9.1 kcal/mol, and the critical result for site 1 was the best, suggesting that SAA interfered with C3b activity by interacting with this site (Figs. 2C and D; Table 1).

**Table 1:** Docking data of SAA and C3b protein

Site	Affinity	Site	Affinity
	(kcal/mol)		(kcal/mol)
site1	-9.1	site6	-6.6
site2	-4.5	site7	-3.3
site3	-6.7	site8	-3.3
site4	-8.5	site9	-7.6
site5	-6.6	site10	-6.8

### In vivo anti-complement effect

**Fig.3:**
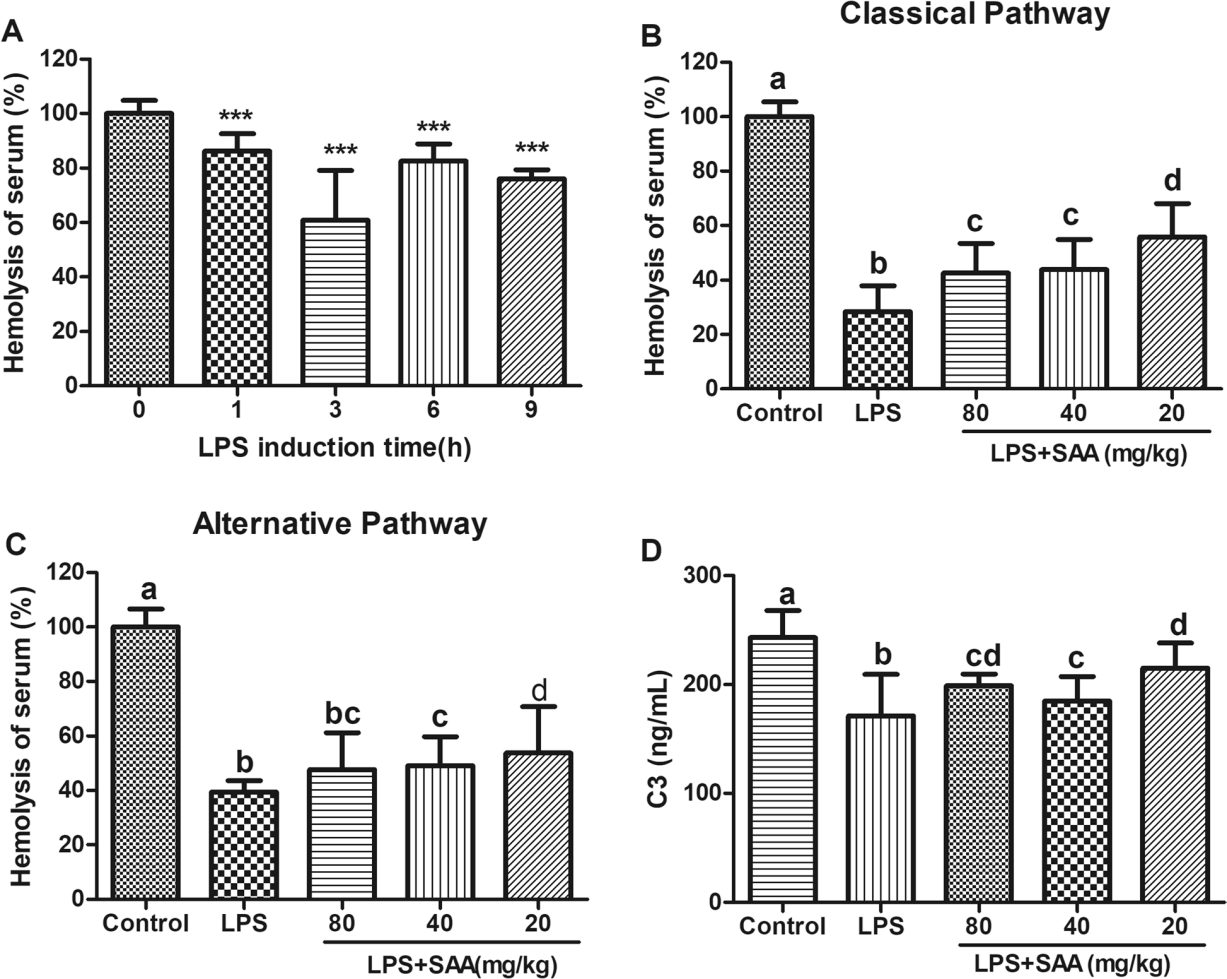
Effect of SAA on complement activity in LPS-induced rats. **A** Hemolytic activity of the serum at LPS induced at different times. Data were listed as mean ± SD (*n* = 4), ***P < 0.001 compared with the 0 h group, statistical tests used were t-test. The hemolytic activity of CP (**B**) and AP (**C**) of the serum was obtained at 3 h. **D** Contents of complement component C3 in the serum were collected at 3 h. Data were presented as mean ± SD (*n* = 5), statistical analysis used were ANOVA (*P*<0.05), letters marked between different groups, if one is the same, the difference is not significant; if they are all different, the difference is significant

The activity of rat complement after LPS tail vein injection was measured by classical hemolysis test. After the LPS injection for 1, 3, 6, and 9 h, a significantly reduced complement activity was observed in the serum (*P* < 0.001), and the 3 h activity was the lowest (Fig. 3A). After the treatment with 80, 40, and 20 mg/kg SAA, the serum complement activity significantly increased (*P* < 0.05 compared with LPS; Figs. 3B and C), and C3 consumption was reduced (Fig. 3D).

### Protective effects of SAA on LPS-induced DIC rats

**Fig.4:**
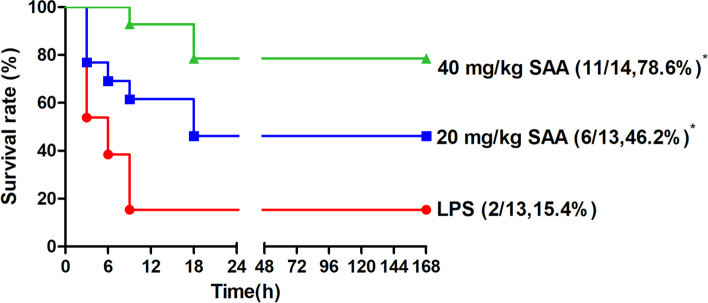
Protective effect of SAA on LPS-induced DIC rats. DIC was induced by LPS. A total of 20 and 40 mg/kg SAA was administered by i.p. injection 1 h after, and 15 mg/kg LPS was administer by intravenous infusion. Survival was monitored for over 168 h. *P < 0.05 compared with the LPS group, statistical tests used were t-test

We investigated the protective effects of SAA on LPS-induced DIC using a rat model. A total of 15.4% (2/13) of the rats infused with LPS survived 24 h after the start of the experiment. SAA treatment for 1 h was performed before LPS induction of DIC. The SAA treatment significantly increased the survival rate (*P* < 0.05; compared with the LPS control group, Fig. 4). A total of 6 out of 13 rats (46.2%) survived in the low-dose SAA treated group, and 11 out of 14 rats (78.6%) survived in the high-dose SAA treated group.

### Effects of SAA on biochemical and pathological damages in LPS-induced DIC rats

To further elucidate the LPS-induced outcome of SAA on DIC, we systematically investigated the biochemical and pathological effects using a rat model. Table 2 summarizes the coagulation indicators of the whole blood detected by thromboelastography in all groups, as follows: coagulation factor activity (R), fibrinogen function (K, angle), platelet function (MA), and CI. The values of coagulation factor activity, fibrinogen function, platelet function, and CI for the LPS-induced DIC rats were all significantly lower than those for normal rats (*P* < 0.05, Table 2). SAA injection significantly attenuated the increased coagulation factor activity, fibrinogen function, and platelet function and decreased CI (*P* < 0.05) in comparison with the LPS group (Table 2). However, 40 mg/kg SAA i.p. injection did not affect the coagulation function of rats (Table 3).

**Table 2:** Effect of SAA on the coagulation function of DIC induced by LPS

Sample	R(min)	K(min)	Angle(°)	MA(mm)	CI
Control	2.21 ± 0.31a	1.11 ± 0.21a	74.39 ± 2.81a	70.02 ± 1.44a	4.568 ± 0.63a
LPS	3.29 ± 0.67b	2.95 ± 1.31b	56.679 ± 9.92b	49.91 ± 7.47b	-0.65 ± 0.75b
LPS + SAA (40 mg/kg)	2.52 ± 0.52a	1.59 ± 0.58a	67.55 ± 6.03a	61.48 ± 6.03c	2.62 ± 1.68c

40 mg/kg SAA was administered by i.p. injection 1 h after, and 15 mg/kg LPS was administered by intravenous infusion. 3 h after, blood was obtained, and the coagulation function was tested with thromboelastometry. Data were presented as the mean ± SD. *n* = 9 in the Control group, *n* = 11 in the LPS group, and *n* = 9 in the LPS + SAA (40 mg/kg) group. Statistical analysis used were ANOVA (*P*<0.05), letters marked between different groups, if one is the same, the difference is not significant; if they are all different, the difference is significant

**Table 3:** Effects of SAA on rats coagulation system

Time	R(min)	K(min)	Angle(°)	MA(mm)	CI
0 h	2.88 ± 0.72a	1.2 ± 0.21a	73.25 ± 2.72a	68.12 ± 2.69a	3.75 ± 0.59a
1 h	3.26 ± 1.25a	1.36 ± 0.24a	69.58 ± 4.97a	68.14 ± 1.30a	3.20 ± 1.25a
2 h	2.65 ± 1.25a	1.33 ± 0.31a	71.57 ± 4.44a	65.08 ± 3.53a	3.42 ± 1.22a
4 h	3.30 ± 0.66a	1.28 ± 0.24a	71.80 ± 3.43a	69.55 ± 4.05a	3.55 ± 0.84a

SAA at 40 mg/kg was injected intraperitoneally. Blood was obtained, and the coagulation function was tested with thromboelastometry. Data were presented as the mean ± SD (*n* = 5). Statistical analysis used were ANOVA (*P*<0.05), letters marked between different groups, if one is the same, the difference is not significant; if they are all different, the difference is significant

**Fig.5:**
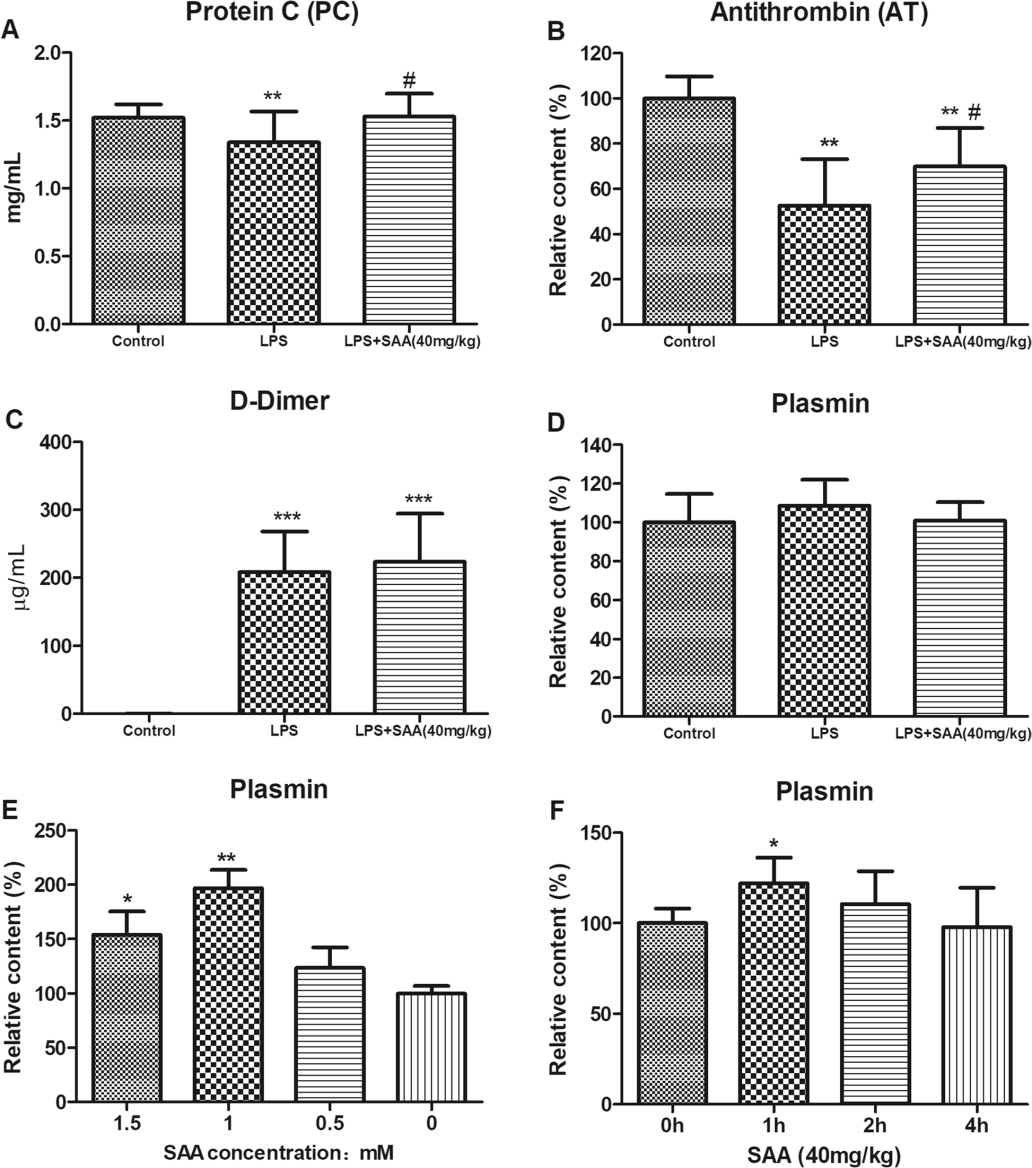
Parameters of SAA on the coagulation in rats with LPS-induced DIC. **A** Protein C contents in plasma. **B** Antithrombin contents in plasma. **C** D-Dimer contents in plasma. **D** Plasmin contents in plasma. **E** SAA and rat whole blood were bathed at 37 °C and centrifuged to obtain the supernatant, and the chromogenic substrate S2251 was used to detect plasmin contents. **F** A total of 40 mg/kg SAA was injected intraperitoneally, and the chromogenic substrate S2251 was used to detect plasma plasmin contents. Data are presented as the mean ± SD. **A**, **B**, **C**, and **D**, *n* = 10, **P < 0.01, and ***P < 0.001 compared with the Control group, ^#^*P* < 0.05 compared with the LPS group. **E** *n* = 5, *P < 0.05, and **P < 0.01 compared with the 0 group. **F** *n* = 5, *P < 0.05, compared with the 0 h group. Statistical tests used were t-tests

The values of protein C and ATIII for the LPS-induced DIC rats were all significantly lower than those for normal rats (*P* < 0.01, Figs. 5A and B, respectively). The values of D-dimer were considerably higher than those for normal rats (*P* < 0.001, Figs. 5C). SAA treatment improved the decreased activities of protein C and ATIII (*P* < 0.05) compared with the LPS group (Figs. 5A and B, respectively). However, SAA treatment had no effect on the increase in D-dimer (Fig. 5C). Incubation of SAA with rat whole blood can significantly increase plasmin activity (*P* < 0.05, Fig. 5E). After the injection of 40 mg/kg SAA intraperitoneally for 1 h, plasmin activity in rats significantly increased. This effect weakened with increasing time and returned to normal level in 4 h (*P* < 0.05, Fig. 5F).

### Inhibition of complement activity by SAA in LPS-induced DIC rats

**Fig.6:**
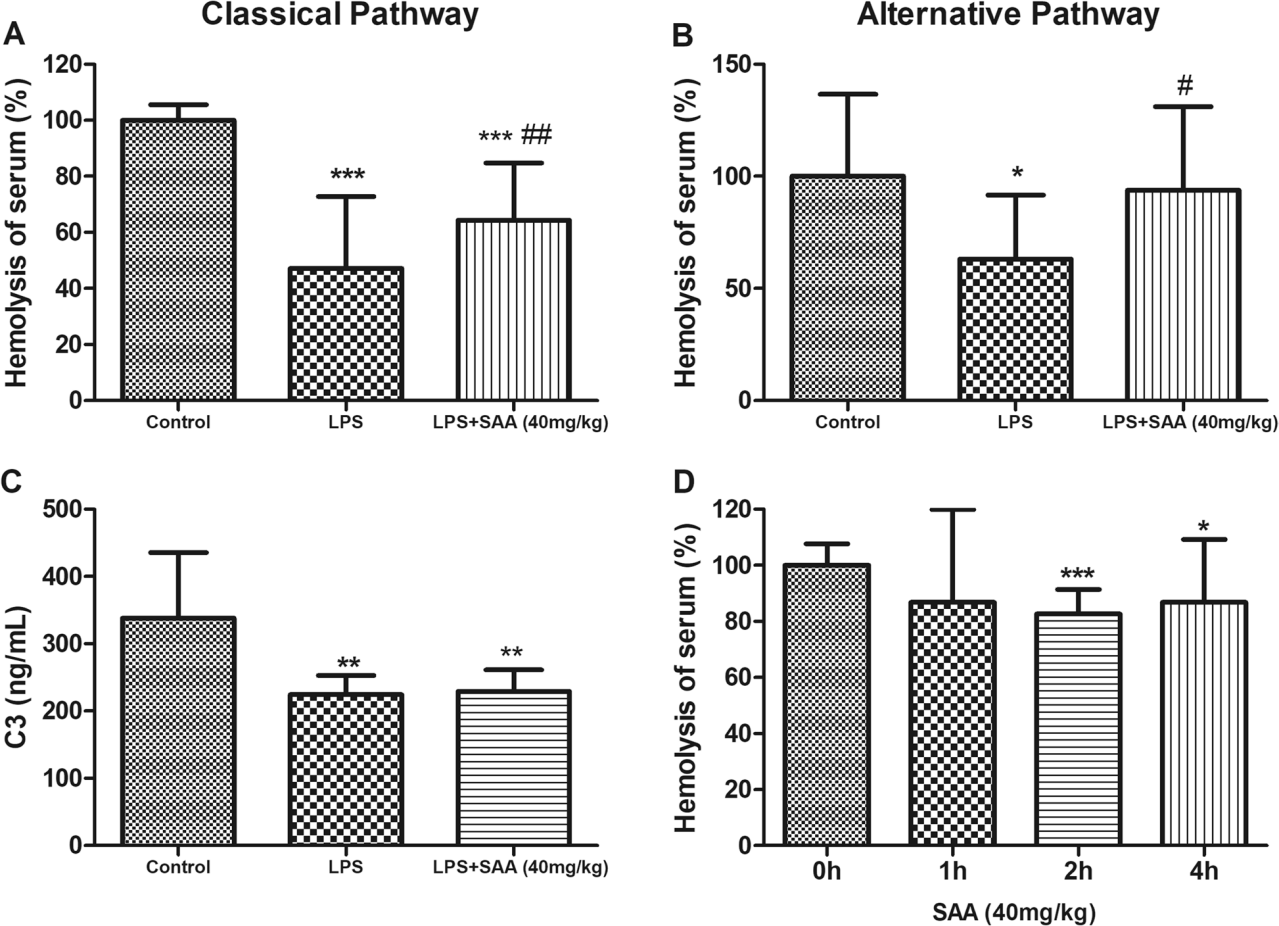
Effects of SAA on the complement activity in LPS-induced DIC. Hemolytic activity of classical pathway (**A**) and alternative pathway (**B**) of the serum. **C** Contents of complement component C3 in serum. **D** Hemolytic activity of the serum under SAA inducement at different times. Data were presented as the mean ± SD. **A**, **B**, and **C**, *n* = 10, *P < 0.05, **P < 0.01, and ***P < 0.001 compared with the Control group, ^#^*P* < 0.05, and ^##^*P* < 0.01 compared with the LPS group. **D** *n* = 5, *P < 0.05, and ***P < 0.001 compared with the 0 h group. Statistical tests used were t-tests

The LPS-induced DIC caused a sharp reduction in serum complement activity, and 40 mg/kg SAA significantly diminished the decrease in serum complement activity (*P* < 0.05, Figs. 6A and B). However, SAA treatment caused no effect on the LPS-induced DIC complement C3 depletion (Fig. 6C). After the injection of SAA intraperitoneally for 2 h, the complement activity in rats was significantly inhibited (*P* < 0.05, Fig. 6D).

### Effects of SAA on blood routine examination and inflammatory cytokine levels in LPS-induced DIC rats

**Fig.7:**
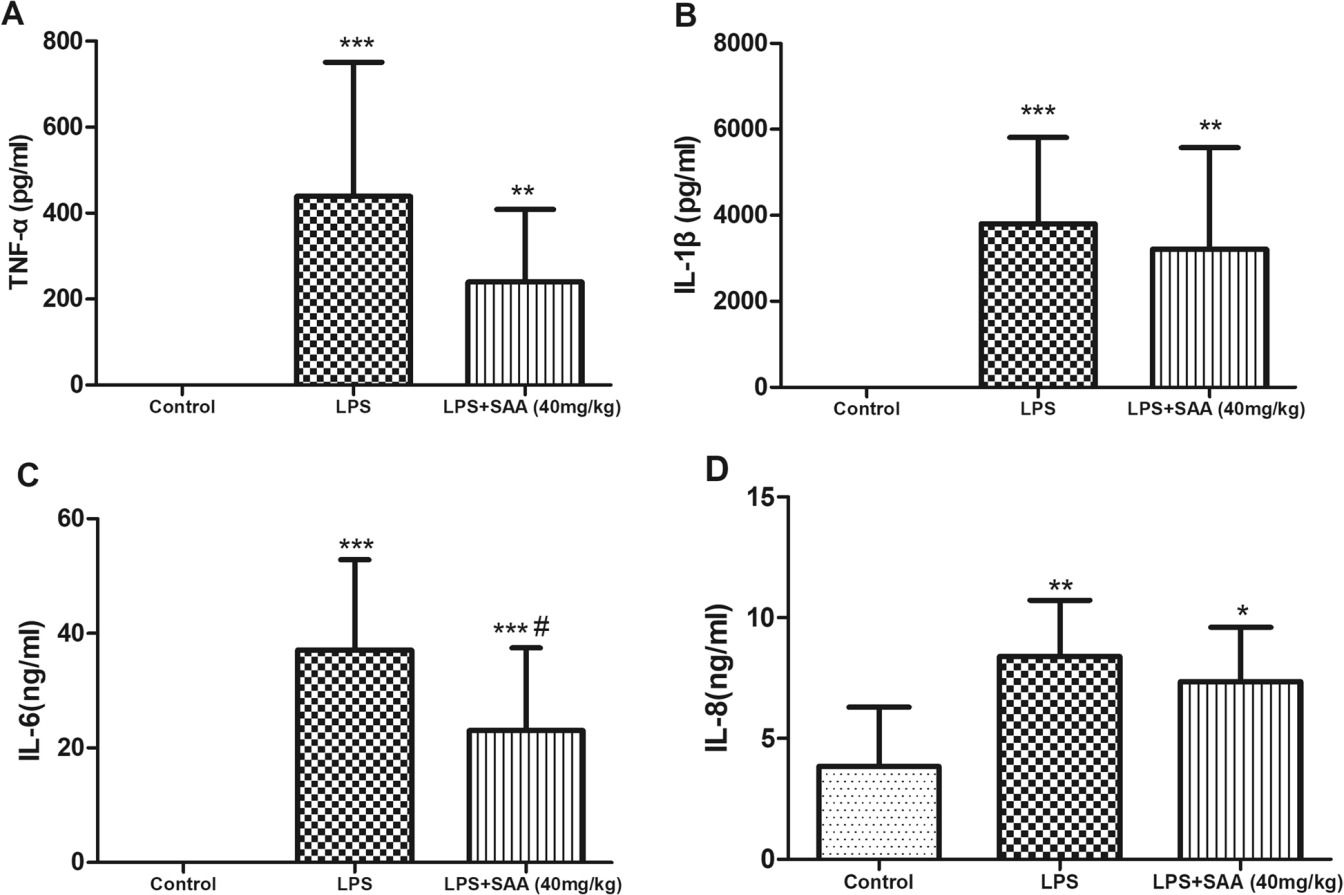
Effects of SAA on inflammatory cytokine levels in LPS-induced DIC. At 3 h after LPS administration, (**A**) TNF-α, (**B**) IL-1β, (**C**) IL-6, and (**D**) IL-8 contents in the serum were detected. Data were presented as the mean ± SD. *n* = 9 in the control group, *n* = 11 in the LPS group, and *n* = 10 in the LPS + SAA (40 mg/kg) group. *P < 0.05, **P < 0.01, and ***P < 0.001compared with the Control group. ^#^*P* < 0.05 compared with the LPS group. Statistical tests used were t-tests

TNF-α, IL-1β, IL-6, and IL-8 increased markedly in the LPS-induced DIC group. The effects of SAA on TNF-α, IL-1β, and IL-8 were marginal, except for a reduction observed for IL-6 (Fig. 7).

### Effects of SAA on the organ injury and function in LPS-induced DIC rats

**Fig.8:**
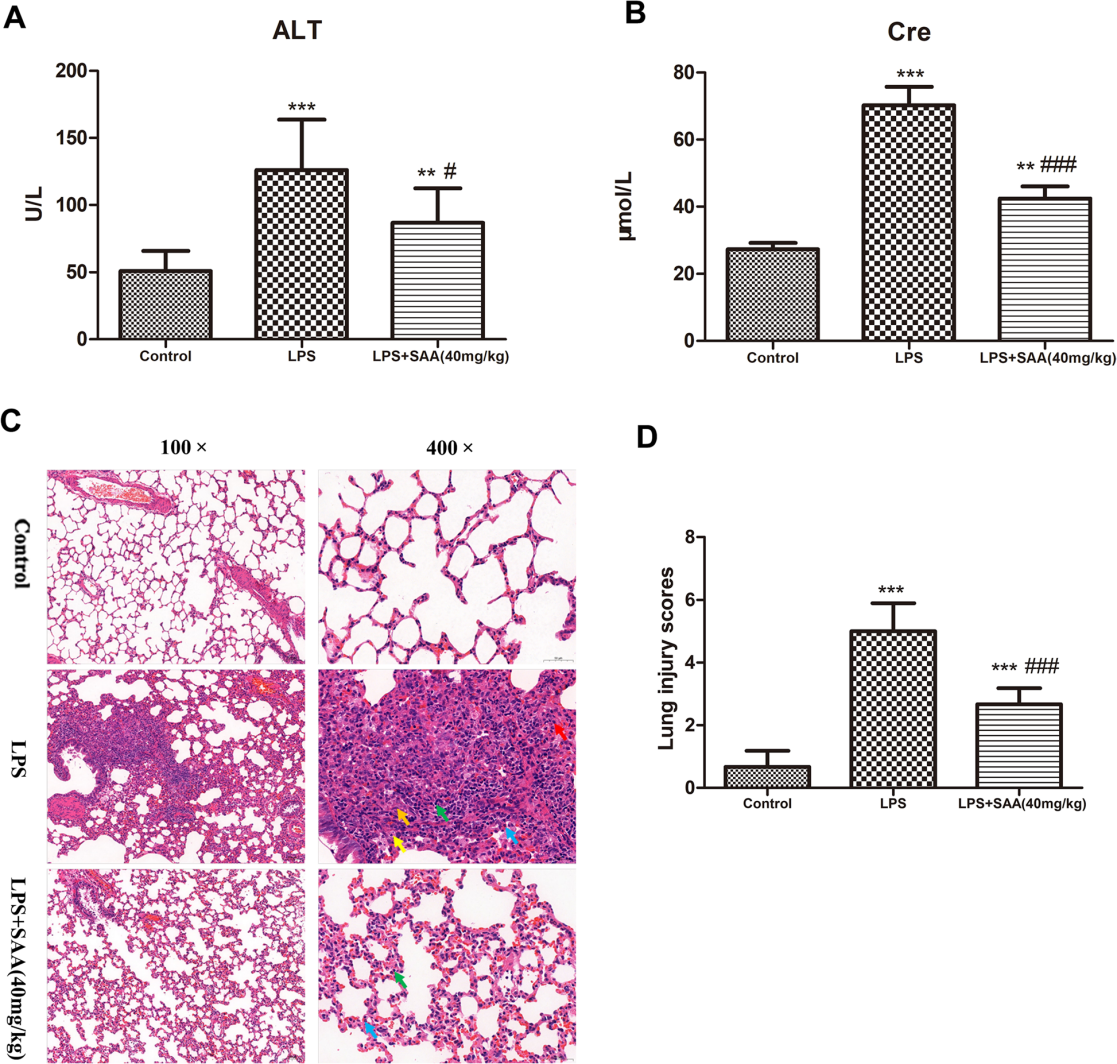
Protective effects of SAA on the organ injury and function in LPS-induced DIC rats. At 3 h after LPS administration, an automatic analyzer was used to detect the ALT (**A**) and Cre (**B**) levels in plasma. Data were presented as the mean ± SD. *n* = 8 in the control group, *n* = 11 in the LPS group, and *n* = 9 in the LPS + SAA (40 mg/kg) group. **P < 0.01, ***P < 0.001 compared with the Control group. ^#^*P* < 0.05, ^###^*P* < 0.001 compared with the LPS group. ALT, alanine aminotransferase. Cre, creatinine. At 3 h after LPS administration, lung histopathology of rats (H&E staining): lung H&E staining (**C**) and lung injury score (**D**). The original magnifications are 100× and 400×. The red, green, blue, yellow, and orange arrows indicate alveolar epithelial cells, lymphocytes, neutrophils, alveolar macrophages, and fibroblasts, respectively. Results are presented as mean ± SD (*n* = 6 in each group). ***P < 0.001 compared with the Control group. ^###^*P* < 0.001 compared with the LPS group. Statistical tests used were t-tests

We investigated the effects of SAA on the liver and renal injuries in LPS-induced DIC rats. The plasma levels of ALT, an indicator of liver injury, were increased by LPS infusion. However, the ALT levels were significantly lower in the SAA treated rats (*P* < 0.05) compared with the LPS group (Fig. 8A). A similar finding was observed in the plasma levels of Cre, which is an indicator of renal injury. An increase in the Cre levels, which SAA significantly suppressed, was observed in the LPS group (*P* < 0.001) compared with the LPS group (Fig. 8B). The results of liver and kidney H&E staining showed that no obvious pathological changes were found in the liver and kidney tissues when the rats were challenged with LPS for 3 h (results not shown).

The lung is the earliest and most easily damaged organ in the pathological process of sepsis, and patients with sepsis can have lung damage in the early stage of the disease course [46–48]. We investigated the effect of SAA on the lung histopathologic change in rats with LPS challenges. The H&E staining revealed that the lung injury of LPS-challenged rat was characterized by alveolar septal thickening, alveolar epithelial cell proliferation and necrosis, and inflammatory cell infiltration. SAA pretreatment alleviated these changes. The results demonstrated that SAA attenuated LPS-induced lung injury (Fig. 8C and D).

## Discussion

**Fig. 9:**
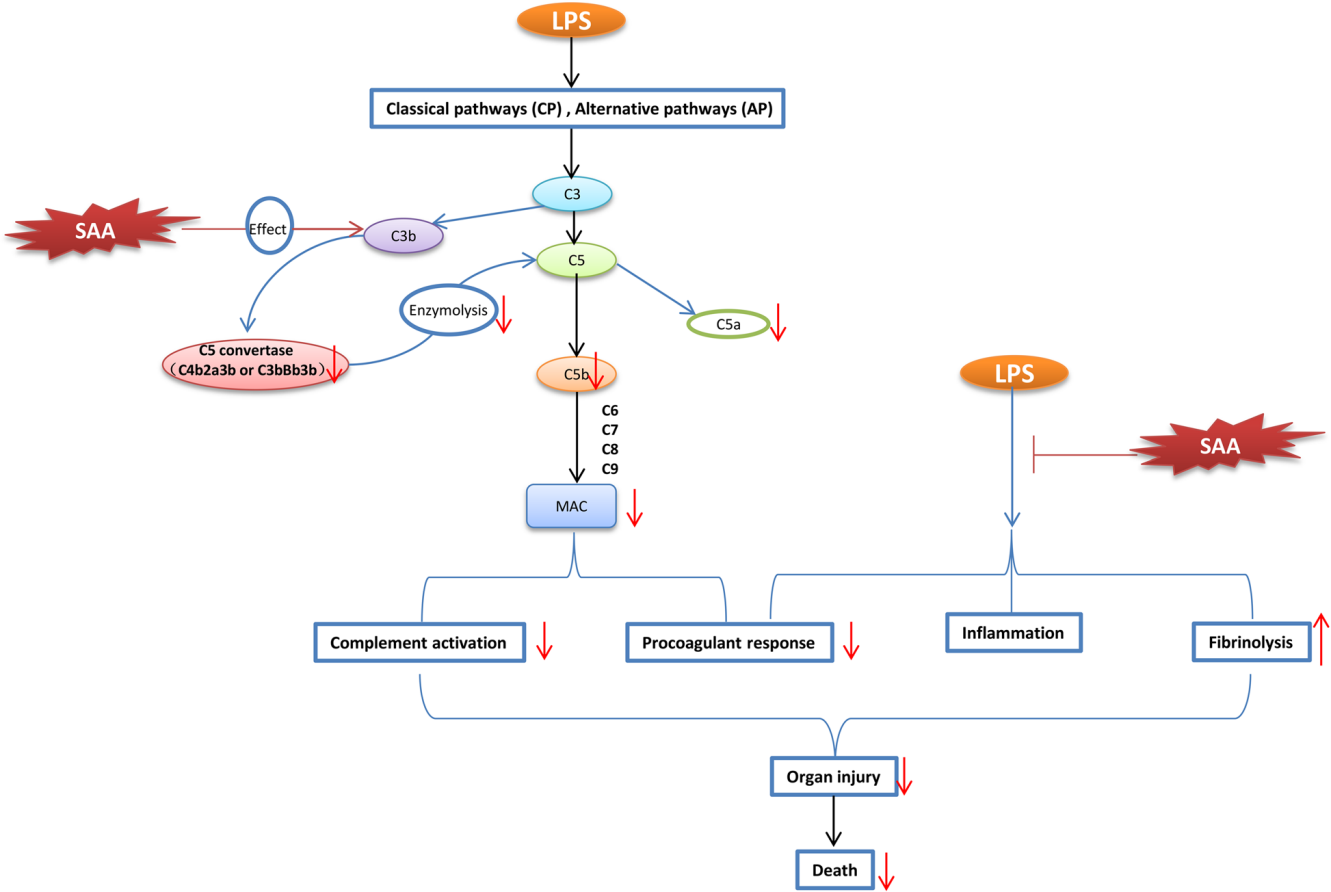
Schematic drawing of pharmacological effects and mechanisms of SAA

We explored the effect of SAA in the alleviation of LPS-induced DIC by inhibiting complement activation. LPS, a constituent of the outer membrane of Gram-negative bacteria, is the most widely used inducer of DIC in animal models and a major pathogenic factor contributing to the initiation of life-threatening DIC [49]. The entrance of LPS to the bloodstream results in the activation of immune and endothelial cells, thereby eventually leading to the activation of the complement and coagulation systems [49, 50]. An infusion of LPS resulted in the typical changes in DIC, as follows: complement system activation; a significant increase in D-Dimer, IL-6, TNF-a, ALT, and Cre; a decrease in complement C3, protein C, and ATIII; a reduction in the levels of clotting factor, fibrinogen, and platelets; and a high mortality rate. SAA may affect the formation or activity of C5 convertase by targeting the complement C3b protein, thereby inhibiting the terminal activation of complement in the CP and AP of the complement. In the LPS-induced rat DIC model, SAA treatment can do the following: block LPS-induced rat complement terminal activation; reduce the consumption of coagulation factors, plasma fibrinogen, and platelets; and reduce the reduction of protein C and ATIII activity. SAA treatment can prevent consumptive coagulation, improve the biochemical indicators of kidney and liver damage, attenuated lung injury, and improve the survival rate of rats (Fig. 9).

SAA showed a significant inhibitory effect on the CP and AP of the complement (Fig. 1). This study investigated its possible mechanism of action based on the results. The C3/C5 convertase formation experiment was used to detect its inhibitory effect on C3/C5 convertase. The sample was incubated with sensitized sheep red blood cells and rat serum to form C3/C5 convertase. The red blood cells were washed and reacted with the added rat serum containing EDTA. Given that the serum-containing EDTA can no longer generate new C3/C5 convertase, only the previously formed C3/C5 convertase can stimulate the hemolysis reaction of the CP of the complement, and the degree of hemolysis was positively correlated with the amount of C3/C5 convertase [34, 35]. SAA significantly inhibited the classical pathway C3/C5 convertase formation and showed a certain dose dependence (Fig. 2A). In the classical hemolysis experiment, C3 in rat serum was completely consumed (Fig. 2B). SAA did not cause the inhibition of the classical pathway C3 convertase formation, which may inhibit the CP and AP common protein C3b. SAA and C3b protein were molecularly docked. The binding energies of 10 potential sites were between -3.3 and -9.1 kcal/mol, and the critical result to site 1 was the best, thereby suggesting that SAA most likely interfered with C3b activity by interacting with this site (Figs. 2C and D; Table 1). However, the limitations of this study included the lack of in-depth study of mechanisms and pathways.

Complement activation in DIC patients is well known; a chain reaction occurs between the complement and the coagulation system [13, 51, 52]. The activation of both the complement and coagulation systems involves assembling proteolytic complexes and converting zymogens. These proteolytic complexes are primarily serine proteases with high substrate specificity; thus, serine proteases in either system may act on the substrate of another system [17, 51–53]. In this study, SAA substantially decreased LPS-induced DIC coagulopathy (Table 2). SAA inhibited complement activity in rats (Fig. 6D) and inhibited LPS-induced complement activation in rats (Figs. 3B, C and  6A, B).

The manifestations of coagulopathy in LPS-induced DIC were from normal to hypercoagulability, hyperfibrinolysis, and hypocoagulability [49]. Compared with the control group, the R and K times of the LPS group were significantly prolonged, whereas the MA value, CI, and α angle were significantly decreased, indicating that the LPS group showed hypocoagulation; moreover, coagulation factor, fibrinogen, and platelet function were significantly decreased (Table 2). SAA reduced LPS-induced DIC coagulation factor, fibrinogen, and platelet consumption and improved hypocoagulation (Table 2). SAA had no direct effect on blood coagulation function in normal rats (Table 3), consistent with the results reported in the literature, which showed that SAA affected platelet function but had no effect on blood coagulation function [31]. SAA cannot prevent the thrombocytopenia caused by LPS-induced DIC (Table 4), which indicated that complement activation upstream of C5 contributed to platelet clearance. SAA improved LPS-induced DIC coagulation disorders, which may be due to the joint action of SAA anti-complement and anti-platelet effects.

**Table 4:** Effects of SAA on blood routine examination in LPS-induced DIC

	Control	LPS	LPS + SAA(40 mg/kg)
WBC(10^9^/L)	4.04 ± 1.61a	2.41 ± 0.91b	2.67 ± 1.35b
Lymph(%)	39.62 ± 11.18a	52.07 ± 14.56b	46.01 ± 7.67ab
Mon(%)	2.68 ± 1.24a	5.94 ± 4.93ab	6.32 ± 3.744b
Gran(%)	57.7 ± 11.21a	41.96 ± 12.24b	47.67 ± 7.57b
RBC(10^9^/L)	7.6 ± 0.43a	8.54 ± 0.61b	8.06 ± 0.91ab
HGB(g/L)	169.67 ± 7.81a	188 ± 12.67b	173 ± 17.55a
HCT(%)	45.19 ± 1.94a	50.19 ± 3.21b	46.08 ± 4.56a
PLT(10^9^/L)	744.88 ± 303.93a	551.27 ± 133.96a	610 ± 214.84a

40 mg/kg SAA was administered by i.p. injection 1 h after, and 15 mg/kg LPS was administered by intravenous infusion. 3 h after, blood was obtained, and the blood routine examination. Data are presented as the mean ± SD. *n* = 9 in the control group, *n* = 11 in the LPS group, and *n* = 10 in the LPS + SAA (40 mg/kg) group. Statistical analysis used were ANOVA (*P*<0.05), letters marked between different groups, if one is the same, the difference is not significant; if they are all different, the difference is significant

Protein C and ATIII are the main anticoagulant proteins in the blood, and their activities can inhibit the coagulation pathway. Activated protein C inactivates activated coagulation factor V, restricts the binding of activated coagulation factor Xa to platelets, and increases the combination of antithrombin and thrombin to enhance anticoagulation. ATIII is a physiological anticoagulant synthesized by endothelial cells, which can bind activated coagulation factors other than coagulation factor V, coagulation factor VII, and coagulation factor VIII to inactivate them [54, 55]. In this study, SAA treatment significantly improved the depletion of protein C and ATIII (Figs. 5A and B). The main reason for the LPS-induced reduction of protein C and ATIII in DIC is the increased thrombin generation leading to substantial depletion rather than decreased production [55, 56]. Treatment with SAA reduces thrombin generation during DIC development and the consumption of coagulation factors, protein C, and ATIII.

Inflammation is an essential factor that accelerates thrombosis formation; the development of thrombosis can exacerbate the severity of inflammation [57]. After SAA treatment, the levels of TNF-α and IL-6 significantly decreased compared with those in the LPS group. However, SAA did not affect the release of IL-8 and IL-1β (Fig. 7). Inhibition of complement has no effect on the release of cytokines, because the cytokines induced by LPS are driven by CD14/Toll-like receptor 4 [58]. SAA suppresses the LPS-induced nuclear factor-κB signaling pathway by targeting IκB kinase β while significantly decreasing the release of serum inflammatory cytokines, according to previous studies [59, 60]. Our results are different from those in the literature possibly because of our single administration; the weak anti-inflammatory capability of SAA in rats was due to its anti-complement effect.

In the fibrinolysis stage of LPS-induced DIC [61], the body produced a large amount of fibrin-related marker (D-dimer) [62]. Compared with the control group, the D-dimer of the LPS and SAA groups significantly increased and showed no change in the plasmin content, and SAA caused no reduction in the D-dimer content of LPS-induced DIC (Figs. 5C and D). Incubation of SAA with rat whole blood can significantly increase plasmin activity in the blood (Fig. 5E). After the injection of 40 mg/kg SAA intraperitoneally for 1 h, the plasmin activity in rats significantly increased (Fig. 5F). SAA increased the plasmin activity and dissolved the microvascular thrombus formed in the early stage of LPS-induced DIC, thereby increasing the content of D-dimer.

Organ failure is one of the most common complications of DIC, and lung, liver, and kidneys are the easily implicated targeted organs in the process of DIC [1]. In LPS-induced DIC rats, increases in serum ALT and Cre, SAA treatment such that ALT and Cre levels obviously decreased (Fig. 8A and B). However, the liver and kidney H&E staining results showed that no obvious pathological changes were found in the liver and kidney tissues when the rats were challenged with LPS for 3 h (results not shown). In the LPS-induced rat model, pathological damage to organs is generally observed after 6 h [63–66]. Therefore, the possible reason is that LPS is only a challenge for 3 h, and DIC is in the early stage, which can detect the biochemical indicators of renal and liver damage, but it has not yet reached the level of histopathological damage. The lung is the earliest and most easily damaged organ in the pathological process of sepsis, and patients with sepsis can have lung damage in the early stage of the disease course [46–48]. We investigated the effect of SAA on the lung histopathologic change in rats with LPS challenges. The lung injury of LPS-challenged rat and SAA pretreatment alleviated LPS-induced lung injury (Fig. 8C and D).

## Conclusion

SAA showed an antagonistic effect on LPS-induced DIC by inhibiting complement activation. The inhibition of complement activation ameliorated coagulation system disorders, controlled the inflammatory reaction, and protected important organs, including the kidney, liver and lung. In the present study, SAA exhibited considerable potential in DIC treatment.

## Data Availability

The datasets used and/or analyzed during the current study are available from the corresponding author upon reasonable request.

## Abbreviations

DIC: Disseminated intravascular coagulation
SAA: Salvianolic acid A
TF: Tissue factor
TNF-α: Tumor necrosis factor-α
IL-1β: Interleukin-1β
IL-6: Interleukin-6
IL-8: Interleukin-8
C3: Complement 3
NHS: Normal human serum
ATIII: Antithrombin III
CP: Classical pathway
AP: Alternative pathway
C3b: Complement 3b
LPS: Lipopolysaccharide
ALT: Alanine aminotransferase
Cre: Creatinine
H&E: Hematoxylin and eosin
